# Antibiotic Susceptibility Profile of Carbapenem Resistant *Escherichia Coli* and *Klebsiella Pneumoniae* in Urinary Isolates from a Tertiary Care Hospital: An Observational Study

**DOI:** 10.31729/jnma.9149

**Published:** 2025-07-31

**Authors:** Gaurav Verma, Shradha Smriti, Rajesh Kumar Dash, Liza Das, Dipti Pattnaik, Nipa Singh, Subhra Snigdha Panda, Sukanta Tripathy

**Affiliations:** 1 Department of Microbiology, Kalinga Institute of Medical Sciences, Bhubaneswar, Odisha, India; 2 Department of Transfusion Medicine, Kalinga Institute of Medical Sciences, Bhubaneswar, Odisha, India

**Keywords:** *antibiotic resistance*, *carbapenem resistance*, *uropathogens*, *nitrofurantoin*, *fosfomycin*

## Abstract

**Introduction:**

Urinary tract infections are common in both hospital and community settings and impose a significant financial burden. Among Enterobacterales, *Escherichia coli* and *Klebsiella pneumoniae* are the leading uropathogens. The rise of carbapenem-resistant strains is concerning due to limited treatment options, with carbapenems often being the last line resort. This study intends to determine the microbiological profile of *E. coli* and *K. pneumoniae* uropathogens obtained from a tertiary care hospital.

**Methods:**

A prospective study was conducted from October 2023 to June 2024 in a tertiary care hospital in Eastern India. Urine samples received for routine culture in the central laboratory were processed following standard microbiological protocols. Bacterial identification and antibiotic susceptibility testing were performed using the VITEK-2 compact system. A total of 958 isolates of *E. coli* and *K. pneumoniae* were obtained. Of these, 198 were identified as carbapenem-resistant as per the Clinical Laboratory Standard Institute 2023 guidelines.

**Results:**

Of the, 7228 urine samples, 4336 (60%) yielded a positive culture. Among these, 1552 (36%) were gram-negative bacteria. Out of the gramnegative bacteria, 1209 (77.8%) were Enterobacterales. *Escherichia coli* were 562 (46.4%) and *Klebsiella pneumoniae* was 396 (32.7%). Carbapenem resistant *E. coli* was 96 (17%) and Carbapenem resistant *K. pneumoniae* was 102 (26%). Both the Carbapenem resistant E. coli and K. pneumoniae showed susceptibility to fosfomycin and nitrofurantoin.

**Conclusions:**

Urinary tract infections caused by *Escherichia coli* and *Klebsiella pneumoniae* are frequent, but Carbapenem resistant strains pose an emerging therapeutic challenge. This study highlights the prevalence and effective antibiotic options.

## INTRODUCTION

Urinary tract infections (UTIs) are among the most common bacterial infections in both community and hospital settings.^1^ While various organisms can cause UTIs, *Escherichia coli* and *Klebsiella pneumoniae* are the predominant uropathogens.^2^ The widespread and often indiscriminate use of antibiotics in humans and animals, along with poor infection control and increased global travel, has contributed to the emergence of multi-drug resistant (MDR) bacteria. Among them carbapenem resistance among uropathogens is a cause of concern as they are last resort treatment for multi-drug resistant bacteria.^1^ A global study reported 4.5% carbapenem resistance among Enterobacterales, while an Indian study noted 5.1% resistance in *E. coli.* ^3,4^ Data from Eastern India remains limited, but a recent study found prevalence of CR *E. coli* and *K. pneumoniae* was 22.73% and 35.29%, respectively.^5^ This study aims to evaluate the prevalence and resistance patterns of CR *E. coli* and *K. pneumoniae* in urine isolates.

## METHODS

This prospective study was conducted from October 2023 to June 2024 in the Department of Microbiology, of a tertiary care hospital. Ethical clearance was obtained from the Institutional Ethics Committee prior to the commencement of the study (reference no. KIIT/KIMS/IEC/1346/2023).

Patients presenting to both outpatient and indoor patients whose urine samples were sent to microbiology laboratory for culture and sensitivity were included in the study. All the bacterial isolates <10^5^ cfu/ml (because this is the clinical threshold for a significant UTIs, counts below this are often considered to be sample contamination rather than active infection), urine collected from catheter and urine bags, mixed bacterial growth, repeated isolates from same patient, contaminants were excluded in the study.

Patients who had been advised for urine culture after clinical examinations were provided with sterile wide-mouth container. The nursing staff/lab technician explained the procedure for collection of urine (mid-stream clean catch urine).^6^ All the patients’ demographic (age, gender) and clinical details (organism isolated) were recorded. The urine samples collected were inoculated on cysteine lactose electrolyte deficient (CLED) agar and the plates were aerobically incubated for 24 hours at 37°C for 18-24 hours. A bacterial colony count of ≥10^5^ colony-forming units (CFU) per milliliter was considered indicative of significant bacteriuria. The identification and antibiotic susceptibility testing was done using VITEK-2 compact system (Biomeriex). The *Enterobacterales* which were found to be resistant to either imipenem, meropenem or ertapenem were considered as carbapenem resistant as per the Clinical Laboratory Institute Standard (CLSI) 2023 guidelines.^7^ Of these only the carbapenem resistant *E.coli* and *K.pneumoniae* were further processed. The isolates which were screened to be resistant to carbapenem antibiotics were phenotypically tested for resistance by Kirby-Bauer disc diffusion method using imipenem 10μg, meropenem 10μg and ertapenem 10μg discs.

AST was performed for isolates, using relevant VITEK antibiotic panels (antibiotic susceptibility panel 235 for *Escherichia coli* and *Klebsiella pneumoniae* (BioMerieux, Durham, NC, USA). Quality control (QC) was performed according to the CLSI 2023 Ml00 guidelines using *E. coli* ATCC 25922 and *K. pneumoniae* ATCC 700603 as reference strains. Control testing was conducted with each new AST panel lot, at regular intervals and during any deviation in expected results. Results were considered valid only if QC strains fell within established susceptibility ranges.

## RESULTS

**Figure 1:**
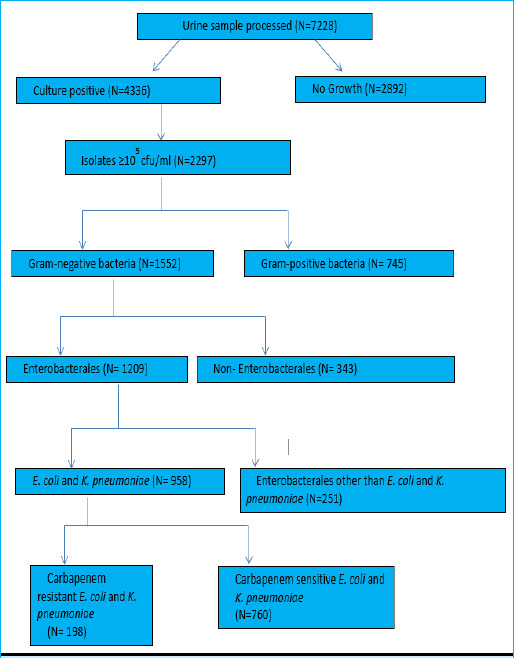
Flow chart of bacterial isolates obtained from urine samples.

A total of 7,228 urine samples processed, 4,336 (60%) were culture positive. Among the culture positive urinary isolates, 1,552 (35.80%) were gram-negative bacteria (GNB). Enterobacterales accounted for 1,209 (77.88%) of the GNB. *E. coli* and *K. pneumoniae* comprised 562 (36.21%) and 396 (25.51%) of the GNB, respectively, together constituting 958 (61.72%) (Figure 1).

There were 562 (36.21%) *E. coli* and 396 (25.52%) *K. pneumoniae* among total GNB urinary isolates (Table 1). 96 (17.08%) *E. coli* and 102 (25.75%) *K. pneumoniae* carbapenem-resistant isolates were obtained.(Table 2).

**Table 1:** Organism wise distribution of gram negative bacterial urinary isolates (n=1552).

Organisms	n(%)
*E. coli*	562(36.21)
*K. pneumoniae*	396(25.52)
*P. aeruginosa*	231(14.88)
*A. baumannii*	112(7.22)
*Enterobacter spp.*	84(5.41)
*Citrobacter spp.*	75(4.83)
*Proteus mirabilis*	31(2.00)
*Salmonella spp.*	24(1.55)
*Providencia spp.*	20 (1.28)
*Serratia spp.*	17(1.10)

**Table 2:** Distribution of carbapenem resistant *E. coli* and *K. pneumoniae* uropathogens (n=198).

Organisms	Total Isolates	Carbapenem Resistant isolates n (%)
*E. coli*	562	96 (17.08)
*K. pneumoniae*	396	102 (25.75)

**Figure 2:**
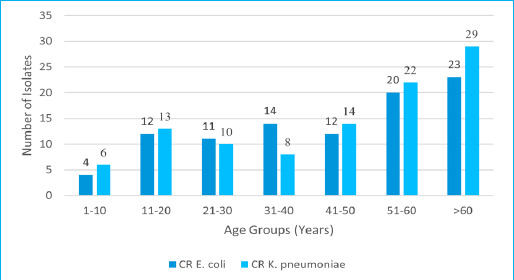
Age wise distribution of carbapenem resistant *E. coli* and *K. pneumoniae* (n=198).

During the study period, 2732 (63.00%) samples were obtained from in-patients department (IPD) and 1604 samples (36.99%) were from out-patients department (OPD). Among IPD samples, carbapenem-resistant *E. coli* and *K. pneumoniae* together accounted for 147 (5.38%), with 62 (2.27%) *E. coli* and 85 (3.11%) *K. pneumoniae.* Among OPD samples, carbapenem-resistant *E. coli* and *K. pneumoniae* together accounted for 51 (3.18%) with 34 (2.12%) *E. coli* and 17 (1.06%) *K. pneumoniae.* There were 106 (53.54%) CR isolates collected from female patients and 23 (23.96%) CR *E. coli,* 29 (28.43%) CR *K. pneumoniae* were isolated from patients over 60 years of age (Figure 2).

**Table 3a:** Antibiotic susceptibility profile of carbapenem resistant *K. pneumoniae* (n=102).

Antibiotics	n(%)
Amoxicillin/Clavulanicacid	1(0.98)
Pipracillin/Tazobactam	0(0)
Ceftazidime	0(0)
Ceftriaxone	0(0)
Ertapenem	0(0)
Imipenem	0(0)
Amikacin	5(4.90)
Gentamicin	3(2.94)
Ciprofloxacin	0(0)
Ofloxacin	0(0)
Fosfomycin	33(32.35)
Nitrofurantoin	19(18.62)
Trimethoprim/sulfamethoxazole	6(5.88)

**Table 3b:** Antibiotic susceptibility profile of carbapenem resistant *E. coli* (n=96).

Antibiotics	n(%)
Ampicillin	1(1.04)
Amoxicillin/Clavulanicacid	0(0)
Pipracillin/Tazobactam	0(0)
Ceftazidime	0(0)
Ceftriaxone	0(0)
Ertapenem	0(0)
Imipenem	8(8.33)
Amikacin	14(14.58%)
Gentamicin	0(0)
Ciprofloxacin	2(2.08)
Ofloxacin	68(70.83)
Fosfomycin	35(36.45)
Nitrofurantoin	5(5.20)
Trimethoprim/Sulfamethoxazole	5(5.20)

Among CR *K. pneumoniae* isolates, 33 (32.4%) showed sensitivity to fosfomycin and 19 (18.6%) showed sensitivity to nitrofurantoin (Table 3a).

Among CR *E. coli* isolates, 68 (70.83%) were sensitivity to fosfomycin, while 35 (36.5%) were sensitivity to nitrofurantoin (Table 3b).

## DISCUSSION

Resistance to carbapenems among uropathogens is of grave concern and we need to address this issue by judicious use of these antibiotics. Urinary tract infections being a common problem affecting large number of patients and carbapenem resistant UTIs are becoming increasingly prevalent and have become a big concern.

In this study *Escherichia coli* (46.5%) and *Klebsiella pneumoniae* (32.7%) were the most prevalent *Enterobacterales* obtained from the urine samples. This finding confers with the findings of various Indian studies like Indian Council of Medical Research (ICMR) Antimicrobial Resistance Surveillance Network 2023 report, Sarita M et al., Smriti S et al., and with other International studies like Cayci Y.T. et al. and Monari C et al. where *E. coli* and *K. pneumoniae* are the predominant *Enterobacterales* isolated from urine samples.^4,8-11^ Singhal et al. have reported *E. coli* to be the most prevalent uropathogen which is in agreement With our findings.^12^

Among the UTIs caused by *Enterobacterales,* approximately 31% are caused by carbapenem resistant bacteria with *E. coli* and *K. pneumoniae* comprising almost 56% and 32.3% of these infections respectively.^13^ In our study CR *E. coli* and *K. pneumoniae* urinary isolates constituted 17% and 26%, respectively. As a whole the prevalence of carbapenem resistance has been reported to be high in various clinical isolates. Study conducted by Modi et al., in 2017 and 2018 reported the prevalence of carbapenem resistant *Enterobacterales* from urine isolates to be 34.7% and 29.3% respectively.^14^ In another study by Perween N et al. the prevalence of CR uropathogens was bit high (36.3%).^15^

Maximum CR *E. coli* and *K. pneumoniae* were obtained from indoor patients (IPD). As per ICMR AMRSN 2023 majority of the E. coli urinary isolates are obtained from OPD while, *K. pneumoniae* are obtained from ward.^9^ Similar findings have also been reported by Smriti S. et al. and Singhal et al.^9,12^ We could not access the data of the OPD patients as the study is conducted in a single tertiary care hospital as OPD patients after consultation either did not send urine samples for culture nor they came for follow up.

In present study there was higher occurrence of CR *E. coli* and *K. pneumoniae* uropathogens, among female patients. Various literature have indicated that UTIs are more prevalent in female patients due to their shorter urethra and close proximity of the urethra to rectal opening which facilitate the entry of bacteria, causing colonization and increasing the risk of UTIs. This finding is consistent with other studies.^9, 11, 16, 17^

Most of the CR *E. coli* and *K. pneumoniae* were obtained from patients aged more than 60 years of age. Ndzime Y. M. et al. have reported that *E. coli* and *K. pneumoniae* causing UTIs are more frequent in individuals aged ≤17 years and those ≥50 years.^18^ In an Indian study it is indicated that in males, higher incidence of UTIs is observed in the age group 40-60 years, which is likely due to increased urinary retention associated with benign prostrate hypertrophy (BPH).^17^ In elderly individuals, the risk of urinary tract infections rises due to age-related immune system decline and reduced functional independence. ^19^

Antibiotic susceptibility testing showed that carbapenem-resistant *E. coli* urinary isolates demonstrated high sensitivity to fosfomycin (71%), followed by nitrofurantoin (36.5%) and gentamicin (14.5%). These findings are in partial agreement with a study by Smriti S. et al., which reported higher sensitivity rates for *E. coli* urinary isolates fosfomycin at 98.6% and nitrofurantoin at 80%. ^9^ Similarly, Sirisha D.T. reported nitrofurantoin sensitivity at 88.4%, while Kalai J. et al. observed a rate of 90%.^20^, ^21^ Both the ICMR AMRSN 2023 and CLSI 2024 guidelines recommend fosfomycin as a preferred treatment for urinary tract infections (UTIs).^7,8^ The susceptibility patterns observed in the current study are consistent with data from other studies in India and internationally, where *E. coli* sensitivity to fosfomycin and nitrofurantoin typically ranges between 80% and 90%. ^9^, ^22^

The antibiotic susceptibility testing of CR *K. pneumoniae* isolates showed increased sensitivity to fosfomycin (32.4%) and nitrofurantoin (18.6%), which are consistent with the findings of a study conducted in Bangladesh Rahman F et al. by where nitrofurantoin showed sensitivity rate of 34.29%. ^23^ Similarly, an international study by Mukubwa et al. reported moderately lower sensitivity rates observed for fosfomycin (59.3%) and a study conducted in Pakistan by Khan et al. found that 78.7% of *K. pneumoniae* urinary isolates were highly sensitive to fosfomycin. ^24, 25^

The limitations of the study is, it is a single tertiary care hospital and did not include molecular analysis of resistance mechanisms and failed to evaluate patient comorbidities or antibiotic history. It was restricted to urinary isolates which limits its broader applicability. Furthermore, the restricted antibiotic susceptibility testing omitted newer treatment options, thereby diminishing the comprehensiveness of resistance profiling and its generalizability to larger healthcare contexts.

## CONCLUSION

There is a growing concern about the shortage of effective antibiotics due to the rise of multidrug-resistant bacteria. Continuous monitoring of antibiotic resistance trends is essential to address this issue. The current study shows that *Escherichia coli* and *Klebsiella pneumoniae* are the most common pathogens causing urinary tract infections and demonstrate highest sensitivity to fosfomycin and nitrofuratoin. Detecting and reporting carbapenem resistance is crucial for improving clinical outcomes by enabling timely optimization of antibiotic selection and initiation of appropriate therapy. To combat this resistance, it is necessary to implement changes in antibiotic consumption patterns and strengthen infection prevention strategies.

## Data Availability

The data are available from the corresponding author upon reasonable request
